# *Ixodes tropicalis* (Acari: Ixodidae) infesting a human and molecular detection of *Rickettsia bellii*, Colombia

**DOI:** 10.7705/biomedica.5464

**Published:** 2021-06-15

**Authors:** Juan C. Quintero, María L. Félix, José M. Venzal, Santiago Nava

**Affiliations:** 1 Grupo de Investigación en Ciencias Veterinarias, Centauro, Facultad de Ciencias Agrarias, Universidad de Antioquia, Medellín, Colombia Universidad de Antioquia Grupo de Investigación en Ciencias Veterinarias, Centauro Facultad de Ciencias Agrarias Universidad de Antioquia Medellín Colombia; 2 Laboratorio de Vectores y Enfermedades Transmitidas, Facultad de Veterinaria, Centro Universitario Regional Litoral Norte, Universidad de la República del Uruguay, Salto, Uruguay Universidad de la República Laboratorio de Vectores y Enfermedades Transmitidas Facultad de Veterinaria, Centro Universitario Regional Litoral Norte Universidad de la República del Uruguay Salto Uruguay; 3 Instituto Nacional de Tecnología Agropecuaria, Estación Experimental Agropecuaria Rafaela, Santa Fe, Argentina Instituto Nacional de Tecnología Agropecuaria Instituto Nacional de Tecnología Agropecuaria Estación Experimental Agropecuaria Rafaela Santa Fe Argentina

**Keywords:** Ixodes, Rickettsia, bacteria, disease vectors, Ixodes, Rickettsia, bacterias, vectores de enfermedades

## Abstract

**Introduction:**

*Ixodes tropicalis* is a little-known tick species reported parasitizing wild rodents only in Colombia and Perú.

**Objective:**

To report a case of *I. tropicalis* infesting a human in the south of the metropolitan area of the Valle de Aburrá, Antioquia, Colombia, and to report the molecular detection of *Rickettsia bellii* in this species.

**Materials and methods:**

The tick was identified using a morphological key and sequencing of tick mitochondrial 16S rRNA. Additionally, bacterial and protozoa pathogens were evaluated using PCR for the detection of *Rickettsia* spp., family Anaplasmataceae, *Borrelia* spp., and piroplasmid.

**Results:**

We identified the tick as an *I. tropicalis* female according to Kohls, 1956, description and to partial 16S rRNA sequences showing a minimum of 5% divergencies c*ompA*red to *Ixodes* sequences. We also detected the *gltA* gene of *R. bellii* in the tick with 99.87% of identity.

**Conclusion:**

This is the first report in Colombia of a species of the *Ixodes* genus parasitizing a human and the first report of the detection of *R. bellii* in this tick species.

## Resumen

**Introducción.:**

*Ixodes tropicalis* es una especie de garrapata poco conocida que se había reportado parasitando únicamente roedores silvestres en Colombia y Perú.

**Objetivo.:**

Reportar un caso de infestación por *I. tropicalis* en un ser humano del sur del área metropolitana del Valle de Aburrá (Antioquia) y reportar la detección molecular de *Rickettsia bellii* en esta especie.

**Materiales y métodos.:**

La garrapata se identificó usando claves morfológicas y mediante la secuenciación de su gen 16S ARNr mitocondrial. Además, se evaluó la presencia de agentes patógenos bacterianos y protozoos usando PCR para la detección de *Rickettsia* spp., la familia Anaplasmataceae, *Borrelia* spp. y piroplásmidos.

**Resultados.:**

La garrapata se identificó como una hembra de *I. tropicalis*, según la descripción de Kohls, 1956, y la secuencia parcial del gen 16S ARNr, la cual mostró una divergencia de mínimo 5 % en la c*ompA*ración con las secuencias de *Ixodes*. Además, se detectó el gen *gltA* de *R. bellii* en esta garrapata con una similitud del 99,87 %. Conclusión. Este es el primer reporte en Colombia de una especie del género *Ixodes* parasitando a un humano y el primer reporte de la detección de *R. bellii* en esta especie de garrapata.

Ticks are non-permanent ectoparasites with a worldwide distribution. With some exceptions, they are obligate hematophagous in both immature and adult stages that infest a great diversity of hosts including amphibians, reptiles, birds, and mammals ^1^. About 950 tick species have been recognized and are included in three families: Agasidae, Nuttalliellidae, and Ixodidae ^2^^-^^4^. Ticks are able to transmit bacteria (spirochetes and *Rickettsia*e), protozoans, viruses, and nematodes, making them one of the most important vectors of pathogenic agents in public and veterinary health ^5^. Hard ticks of the Ixodidae family are also the host species of *Rickettsia* spp. of unknown pathogenicity such as *Rickettsia bellii*^6^. This species has been detected in several species of *Amblyomma* and *Ixodes* genera ^7^^-^^10^.

*Ixodes tropicalis* Kohls, 1956, was described from females collected from the wild rodents Thomasomys nicefori (as *Thomasomys aureus*) in Valdivia (Antioquia), and from *Dactylomys boliviensis* in San Juan, Tambopata, Sandia (Puno, Perú) ^11^. Later, immature ticks determined as *I. tropicalis* were reported infesting another wild rodent, *Nephelomys childi* (as *Oryzomys albigularis*), in the Valle de Pichindé (Valle del Cauca), and the Pichindé virus was isolated from them ^12^. However, this report of *I. tropicalis* should be considered doubtful because its larvae and nymph have not been formally described ^2^. Thus, the only *bona fide* records of *I. tropicalis* correspond to those of the original description ^11^.

This study aims to report a case of *I. tropicalis* infesting a human, as well as the molecular detection of *R. bellii* in the south of the metropolitan area of Valle de Aburrá (Antioquia).

## Materials and methods

On March 18, 2018, a 59-year-old man was gardening at his house in La Tablaza, La Estrella (Antioquia) (6°07'02”N, 75°38'14''W; 1756m). Later, he was found parasitized by a tick in the abdomen umbilical region, which was removed, placed in 96% ethanol, and sent to *Universidad de Antioquia*. The classification of the tick was made following the description of Kohls, 1956 ^11^, with a stereomicroscope (Nikon SMZ1000™, Tokyo, Japan).

For molecular studies, the tick was longitudinally bisected using sterile scalpel blades and forceps, rinsed with distilled water to remove ethanol, and crushed with a homogenization pestle. The DNA was extracted using the commercial kit PureLink Genomic DNA Mini Kit™ (Invitrogen, Germany) following the manufacturer's instructions. DNA was tested by polymerase chain reaction (PCR) targeting the tick mitochondrial 16S rRNA gene ^13^ and *gltA* and *ompA* genes for *Rickettsia* spp., 16S rRNA gene of the family Anaplasmataceae, flagellin gene of *Borrelia* spp., and 18S rRNA gene of piroplasmid ^14^^-^^18^.

## Results

**Figure 1:**
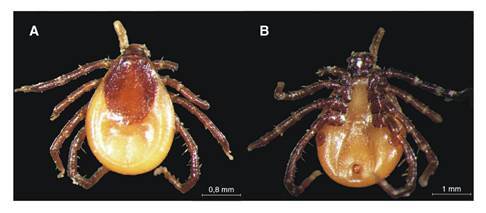
Female of *Ixodes tropicalis*. A. Dorsal view; capitulum, porose areas large, and semicircular in shape. B. Ventral view; hypostome, broken at the base, coxa I with moderately long internal spur, and spiracular plate subcircular in shape. The specimen has been deposited in the “Tick Collection of Instituto Nacional de Tecnología Agropecuaria, Estación Experimental Agropecuaria INTA Rafaela”: (INTA2470).

The tick (a slightly engorged specimen) was identified as a female of *I. tropicalis* based on the following morphological characteristics: Idiosoma suboval, length from the tip of scapulae to the posterior margin of the body (excluding capitulum) 2.15 mm, width 1.66 mm; scutum, with numerous punctations, length 1.30 mm and width 1.15 mm; elevated lateral carinas extending from the scapulae to about the mild-length of the scutum; capitulum, porose areas large and semicircular in shape, separated by about the diameter of one, cornua short and rounded, palpal segment two a little longer than segment three, auricula large and posterolaterally directed; hypostome, broken at the base; coxa I of legs with moderately long internal spur and coxae I-IV with a conspicuous external spur; spiracular plate subcircular in shape (figure 1 A, B).

Photography by José Manuel Venzal

We amplified fragments of the mitochondrial 16S rRNA gene of the tick and *gltA* gene of *Rickettsia* and purified the amplicons using a PureLink Quick PCR Purification Kit^TM^ (Invitrogen, Germany), which we sent to Macrogen (Seoul, Korea) for sequencing. We did not amplify the DNA of piroplasmid, *Borrelia* spp., Anaplasmataceae agents, and the *ompA* gene of *Rickettsia*. The partial sequence obtained for the 16S rRNA gene of the specimen determined as *I. tropicalis* (ca. 410 bp) diverged by more than 5% when compared to the remaining *Ixodes* sequences available at the Genbank.

The partial *gltA* (784 bp) sequence showed 99.87% (783/784 bp) of identity with the corresponding *R. bellii* sequences (GenBank accession numbers: CP000087, AY375161, U59716). The sequences generated in the study were deposited in the GenBank under the accession numbers MT158325 for the 16S rRNA gene of *I. tropicalis* and MT174170 for the *gltA* gene of *R. bellii*.

## Discussion

Besides *I. tropicalis*, another ten species belonging to the genus *Ixodes* are currently recognized in Colombia: *Ixodes affinis* in Carnivora and Artiodactyla ^19^^,^^20^; *Ixodes* auritulus in Passeriformes ^21^; *Ixodes* bocatorensis in Rodentia ^22^; *Ixodes boliviensis* in Didelphimorphia and Carnivora ^20^^,^^23^^,^^24^; *Ixodes montoyanus* in Artiodactyla ^25^^,^^26^; *Ixodes lasallei* in Rodentia ^22^^,^^26^^,^^27^; *Ixodes luciae* in Didelphimorphia ^28^; *Ixodes pararicinus* in Artiodactyla ^19^^,^^29^; *Ixodes tapirus* in Perissodactyla ^11^, and *Ixodes venezuelensis* in Rodentia ^30^. The records of *Ixodes fuscipes*^31^ and *Ixodes brunneus*^23^ for Colombia are currently considered not valid because the taxonomic status of the specimens assigned to these taxa is undetermined ^32^^,^^33^.

Most of these species do not infest humans. Only *I. boliviensis*, *I. brunneus*, and *I. pararicinus* were ocasionally found infesting humans ^34^. For Colombia, ten species of hard ticks have been reported parasitizing humans ^34^^-^^37^: *Amblyomma dissimile, A. mixtum, A. oblongoguttatum, A. ovale, A. patinoi, A. sabanerae, Dermacentor imitans, D. nitens, Rhipicephalus microplus*, and *R. sanguineus sensu lato*. Therefore, this finding corresponds to the first report of the genus *Ixodes* parasitizing humans in Colombia, as well as the first record for *I. tropicalis* in humans.

Regarding the detection of *R. bellii* in Colombia, Miranda, *et al*. (2014), detected it in the free-living larvae of *Amblyomma* sp. ^38^ from the northern coast of Colombia (Los Córdobas, Córdoba). In an area near Los Córdobas, *R. bellii* in *A. ovale* was detected and collected from a donkey in Necoclí ^39^. Besides, *R. bellii* has been detected in larvae of *A. dissimile* collected in *Rhinella horribilis* and *Basiliscus basiliscus* in the department of Magdalena ^40^^,^^41^.

As far as we know, this is the first report of *I. tropicalis* infesting a human and of *R. bellii* in this species in Colombia, and it would broaden the panorama regarding tick species infesting humans and the exposition to *Rickettsia*l agents in the population living in the south of the metropolitan area of the Valle de Aburrá in Antioquia.

These findings demonstrate the presence of *I. tropicalis* as a potential parasite in humans in the south of the metropolitan area of the Valle de Aburrá Valley, as well as the report on the presence in this tick species of *R. bellii*, a bacteria of unknown pathogenicity in humans. Finally, it is crucial to determine other regions at risk of rickettsial agents' transmission besides those already known such as the Urabá area in Antioquia and the Villeta municipality in the department of Cundinamarca.
